# Supplementary material for: Intact ribosomes drive the formation of protein quinary structure
Source: PLoS One. 2020 Apr 24;15(4):e0232015. doi: 10.1371/journal.pone.0232015 (PMC7182177; doi:10.1371/journal.pone.0232015)
Supplement: S1 Fig — (PDF) [file pone.0232015.s001.pdf]

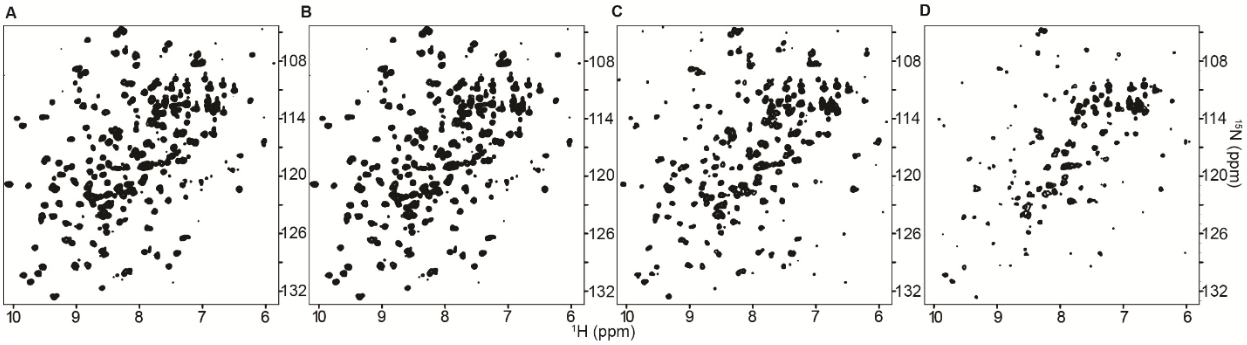

Figure S1. Increasing concentration of ribosomes broadens NMR spectral crosspeaks. ^{1}H-^{15}N HSQC NMR spectra of 10 μM purified [*U*- ^{15}N] γD-crystallin in buffer containing 50 mM NaCl. A) No ribosomes. B) 2 μM ribosomes; C) 4 μM ribosomes; D) 6 μM ribosomes. The data were collected at RT on a Bruker Avance II spectrometer operating at a ^{1}H frequency of 700 MHz with 512 scans per transient; 1024 and 128 complex points were collected in ^{1}H and ^{15}N dimensions and multiplied by a cosine-bell window function and zero-filled to 2048 and 256 points prior to Fourier transformation. The corresponding sweep-widths were 12 ppm and 35 ppm in ^{1}H and ^{15}N dimensions, respectively.
