# Supplementary material for: Intact ribosomes drive the formation of protein quinary structure
Source: PLoS One. 2020 Apr 24;15(4):e0232015. doi: 10.1371/journal.pone.0232015 (PMC7182177; doi:10.1371/journal.pone.0232015)
Supplement: S2 Fig — (PDF) [file pone.0232015.s002.pdf]

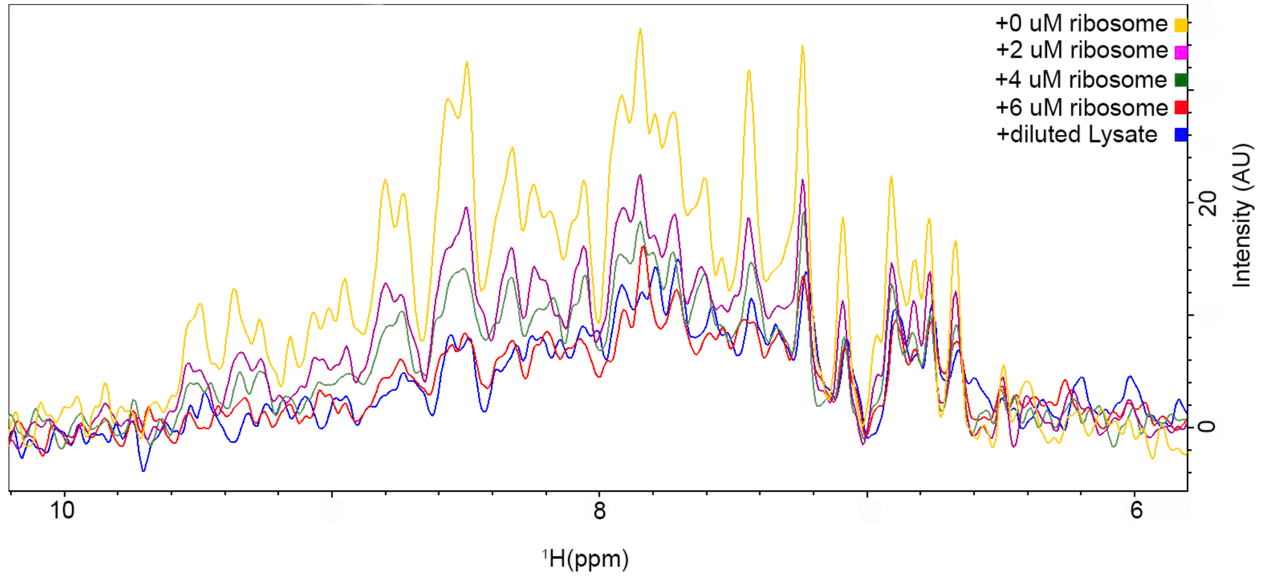

Figure S2. Peak broadening of the ^{1}H-^{15}N HSQC spectrum of [*U*- ^{15}N] γD-crystallin with diluted lysate is consistent with the presence of ~ 5 μM of ribosomes in the sample. Overlay of the amide envelopes of ^{1}H-^{15}N HSQC NMR spectra of 10 μM purified [*U*- ^{15}N] γD-crystallin in buffer containing no ribosomes (yellow); 2 μM ribosomes (purple); 4 μM ribosomes (green); 6 μM ribosomes (red); diluted lysate (blue). The data were collected at RT on a Bruker Avance II spectrometer operating at a ^{1}H frequency of 700 MHz with 512 scans per transient; 1024 and 128 complex points were collected in ^{1}H and ^{15}N dimensions. The first transient of each spectrum was extracted by using "rser 1" command and multiplied by a cosine-bell window function and zero-filled to 2048 points prior to Fourier transformation. The corresponding sweep-widths were 12 ppm and 35 ppm in ^{1}H and ^{15}N dimensions, respectively.
