# Supplementary material for: Intact ribosomes drive the formation of protein quinary structure
Source: PLoS One. 2020 Apr 24;15(4):e0232015. doi: 10.1371/journal.pone.0232015 (PMC7182177; doi:10.1371/journal.pone.0232015)
Supplement: S1 Table — (PDF) [file pone.0232015.s004.pdf]

Table S1. Normalized ^{1}H-^{15}N HSQC peak intensities of γD-crystallin titrated with ribosomes.

RESIDUE #	NO RIBOSOME	2 μM RIBOSOME	4 μM RIBOSOME	6 μM RIBOSOME
**2**	1.518	1.079	1.322	0.957
**3**	0.985	0.708	0.616	0.688
**4**	1.656	1.353	1.497	1.230
**5**	1.034	0.849	0.844	0.601
**6**	0.826	0.694	0.784	0.443
**7**	0.826	0.789	0.990	0.706
**8**	1.860	1.594	1.946	1.371
**9**	0.970	0.832	0.750	0.473
**10**	0.646	0.506	0.347	0.383
**11**	1.612	1.235	1.871	1.530
**12**	0.562	0.420	0.977	0.744
**13**	1.522	1.112	1.508	1.288
**14**	0.994	0.779	0.962	0.708
**15**	0.098	0.014	-0.052	-0.125
**16**	1.473	1.352	0.958	0.637
**17**	0.993	0.826	0.813	0.612
**18**	1.181	1.043	0.901	0.670
**19**	0.681	0.484	0.332	0.634
**20**	1.740	1.563	1.680	1.030
**21**	1.942	1.595	2.792	2.755
**22**	-0.130	-0.027	0.089	-0.089
**23**	0.873	0.852	0.802	0.642
**24**	0.441	0.308	0.417	-0.102
**25**	1.288	1.039	1.201	0.888
**26**	0.186	0.123	0.039	0.216
**28**	0.644	0.389	0.815	0.373
**29**	1.061	0.891	1.150	0.809
**30**	1.019	0.894	0.785	0.499
**31**	2.246	1.824	2.008	1.458
**32**	1.087	0.976	1.057	0.791
**33**	1.455	1.356	1.219	0.987
**34**	0.875	0.627	1.062	0.538
**35**	0.818	0.932	0.650	0.769
**36**	1.820	1.301	2.087	1.376
**37**	1.134	0.818	0.594	0.819
**38**	0.762	0.780	0.701	0.441
**39**	1.102	0.681	0.778	0.594
**40**	0.959	0.754	0.740	0.571

**41**	-0.020	-0.124	0.062	0.141
**42**	1.321	1.176	1.168	0.510
**43**	1.038	0.872	0.616	0.626
**44**	0.744	0.542	0.708	0.499
**45**	0.071	-0.025	0.535	0.420
**46**	0.793	0.577	0.638	0.255
**47**	1.403	1.096	1.339	0.920
**49**	0.696	0.768	0.376	0.604
**50**	2.185	1.878	2.342	2.319
**51**	0.762	0.751	0.776	0.788
**52**	1.312	1.104	1.118	0.970
**53**	1.453	1.045	1.445	1.239
**54**	1.076	0.756	1.169	0.840
**55**	0.958	0.645	0.658	0.255
**56**	0.798	0.522	0.589	0.529
**57**	0.944	0.780	0.814	0.548
**58**	1.113	0.804	0.461	0.584
**59**	0.198	0.057	0.157	0.089
**60**	0.786	0.515	0.538	0.195
**61**	1.255	1.070	1.185	1.484
**62**	1.868	1.460	1.853	1.168
**63**	0.976	0.561	0.768	0.768
**64**	0.767	0.616	0.886	0.923
**65**	1.814	1.421	2.196	1.821
**66**	1.753	1.287	1.587	1.335
**67**	1.388	0.889	2.114	2.148
**68**	1.075	0.775	1.050	0.832
**69**	1.334	1.039	1.142	1.044
**70**	0.916	0.648	0.605	0.354
**71**	0.865	0.513	0.446	0.203
**72**	1.309	1.120	1.483	1.610
**73**	2.276	1.887	1.755	1.345
**74**	1.705	1.426	1.523	1.240
**75**	0.798	0.547	0.732	0.358
**76**	1.025	0.781	0.832	0.594
**77**	1.113	0.870	0.731	0.706
**78**	0.590	0.574	0.406	0.347
**79**	0.659	0.742	0.402	0.427
**80**	0.775	0.524	0.580	0.256
**81**	0.528	0.308	0.308	-0.129
**83**	1.398	0.709	1.450	1.249
**84**	0.819	0.445	0.972	0.360
**85**	0.289	0.139	0.237	-0.097

**86**	3.574	3.036	3.614	3.225
**87**	1.425	1.192	1.703	1.288
**88**	1.143	0.925	0.933	0.778
**89**	1.252	0.994	1.111	0.573
**90**	0.926	0.723	0.751	0.673
**91**	1.891	1.473	1.286	0.820
**92**	1.336	0.919	1.595	1.023
**93**	0.663	0.477	0.347	0.492
**94**	1.161	1.260	1.040	0.455
**95**	0.127	0.130	0.383	0.495
**96**	0.815	0.685	0.665	0.597
**97**	0.764	0.688	0.553	0.166
**98**	0.485	0.551	0.725	0.467
**99**	1.817	1.242	1.375	1.406
**100**	0.882	0.816	0.608	0.190
**101**	0.718	0.277	1.054	1.225
**102**	0.832	0.731	1.128	0.842
**103**	0.318	0.253	0.438	0.302
**104**	0.014	0.092	0.209	0.197
**105**	1.071	0.838	1.107	0.590
**106**	-0.133	-0.137	-0.087	0.338
**107**	0.806	0.748	0.825	0.525
**108**	0.734	0.576	0.372	0.156
**109**	2.097	1.736	1.507	1.260
**110**	0.905	0.912	1.138	0.975
**111**	0.756	0.513	0.862	0.412
**112**	1.535	1.476	1.665	1.097
**113**	0.324	0.039	0.042	0.235
**114**	1.687	1.230	1.918	1.122
**115**	0.422	0.616	0.612	0.538
**116**	0.007	-0.170	0.157	0.299
**117**	0.667	0.329	1.856	0.642
**118**	0.514	0.594	0.711	0.509
**119**	2.365	1.812	2.589	1.815
**120**	0.143	0.317	-0.127	0.102
**121**	1.011	0.734	0.912	0.619
**122**	1.359	1.154	1.567	0.717
**123**	0.936	0.683	0.728	0.595
**124**	1.039	0.929	0.802	0.371
**125**	0.994	0.833	0.779	0.708
**126**	0.716	0.575	0.631	0.424
**127**	2.208	1.654	2.308	1.509
**128**	1.028	0.755	0.763	0.246

**129**	1.232	0.897	1.188	0.469
**130**	1.022	0.860	0.610	0.548
**131**	0.892	0.885	0.747	0.475
**132**	0.699	0.484	0.809	0.575
**133**	0.997	0.886	0.662	0.370
**134**	1.035	0.742	0.661	0.667
**135**	1.447	1.560	1.280	0.966
**136**	1.330	1.142	1.036	0.997
**137**	0.744	0.694	0.696	0.487
**138**	1.971	1.778	1.648	0.740
**139**	0.620	0.499	0.994	0.790
**140**	1.125	0.980	1.133	0.803
**141**	0.745	0.577	0.556	0.753
**142**	1.089	0.767	0.873	0.668
**143**	1.262	1.023	1.059	0.393
**144**	0.961	0.792	0.892	0.441
**145**	0.922	0.704	0.597	0.466
**146**	1.221	0.811	1.120	0.775
**148**	0.574	0.414	0.125	-0.036
**149**	1.589	1.333	1.615	1.410
**150**	1.263	1.143	1.161	0.540
**151**	1.715	1.373	1.883	1.486
**152**	1.177	0.923	1.186	1.008
**153**	-0.585	-0.622	-0.698	0.036
**154**	1.417	1.263	1.507	1.202
**155**	3.604	2.722	3.365	2.899
**156**	2.262	1.636	2.581	1.767
**157**	0.793	0.658	0.871	0.825
**158**	1.276	0.930	1.428	0.827
**159**	1.470	1.184	1.236	0.966
**160**	1.688	1.315	1.574	1.413
**161**	1.331	1.053	1.444	1.261
**162**	2.921	2.415	2.128	2.070
**163**	0.370	0.109	0.080	0.525
**164**	0.739	0.479	0.431	0.370
**165**	0.992	0.955	0.960	0.590
**166**	0.450	0.512	0.670	0.625
**167**	0.226	0.254	0.372	0.336
**168**	1.030	0.868	0.933	0.924
**169**	1.034	0.940	1.399	1.129
**170**	0.774	0.777	0.922	0.446
**171**	-0.013	-0.043	-0.116	0.003
**172**	1.271	1.341	1.756	1.287

**173**	2.765	2.762	4.555	5.077
