# Supplementary material for: Intact ribosomes drive the formation of protein quinary structure
Source: PLoS One. 2020 Apr 24;15(4):e0232015. doi: 10.1371/journal.pone.0232015 (PMC7182177; doi:10.1371/journal.pone.0232015)
Supplement: S1 Raw images — (PDF) [file pone.0232015.s005.pdf]

Raw Image
for Figure 2A

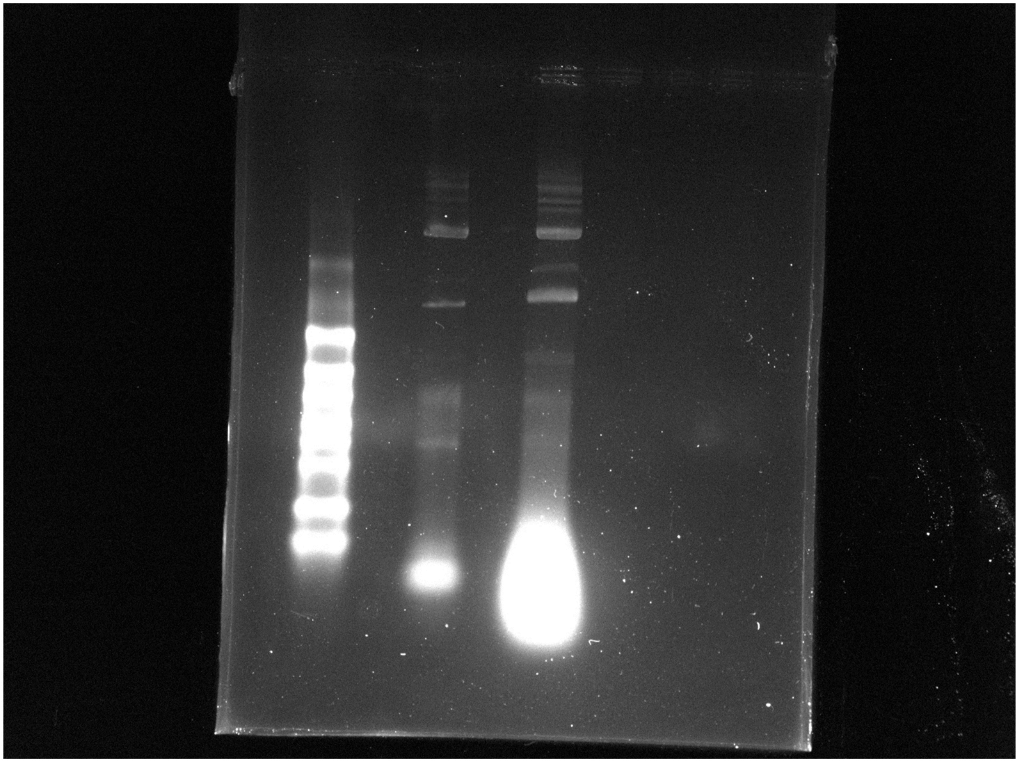

Raw Image
for Figure 2B

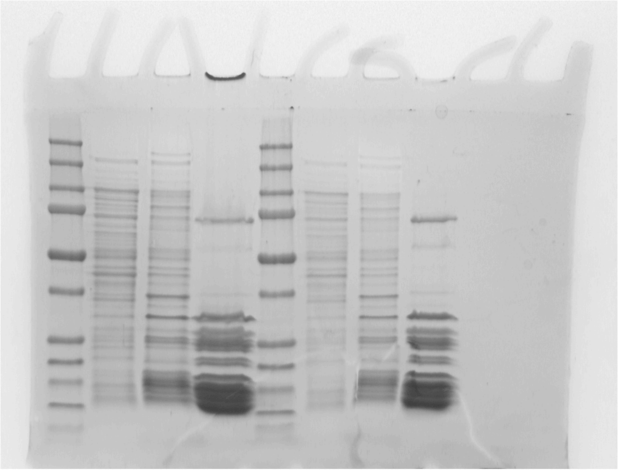

Biorad gel Imaging system was used to capture the gel images
