# Supplementary material for: Intact ribosomes drive the formation of protein quinary structure
Source: PLoS One. 2020 Apr 24;15(4):e0232015. doi: 10.1371/journal.pone.0232015 (PMC7182177; doi:10.1371/journal.pone.0232015)
Supplement: Attachment — Submitted filename: Response to Reviewers.pdf [file pone.0232015.s006.pdf]

Response to Reviewers'

We thank our editor and reviewers for the comments. We addressed all of the concerns raised by the editor and reviewers. In addition, based on the comments, we revised the text throughout to emphasize the purpose of these experiments, *i.e.* to demonstrate that the ribosome must remain intact to affect quinary interactions, and that ribosome-protein interactions are a generalized phenomenon.

Editor:

In the current version, the manuscript suffers from major shortfalls as indicated by the reviewers. These include 1. relevance (gamma-D-crystalline is not a relevant protein to study soft interactions with the E.coli ribosomes). It is not clear why the authors chose to do the experiments using gamma-D-crystalline, and they don't give any rational explanation for this.

We included the rationale for studying γD-crystallin in *E. coli* in the first results section. "To demonstrate that intact ribosomes are a critical component of quinary interactions, the NMR spectrum of purified uniformly labeled [*U*- ^{15}N] γD-crystallin was examined in the presence of stable and destabilized ribosomes in *E. coli* cell lysate. γD-crystallin is a small, 21 kDa, eukaryotic protein found in the eye lens of vertebrates. The protein was studied in *E. coli* lysate to provide an experimental environment that was devoid of specific binding interactions that could obscure the effects of RPIs. Since quinary interactions are transient, they are not expected to interfere with high affinity interactions involved in ribosomal function. Consequently, the effect of the binding interaction on the activity γD-crystallin or the ribosome was not considered in these experiments".

2. *How do the authors reach the conclusion that non-specific electrostatic interactions are formed between gamma-D-crystalline and the ribosome. While they provide a number of references, they did not measure the salt effect on line broadening. Moreover, the E.coli cytoplasm has an ionic strength of ~200 mM. Would line broadening be observed in the E.coli cytoplasm?*

First, we added the in-cell NMR spectrum of γD-crystallin in *E. coli* (Fig 1B), which shows extensive line broadening. Second, we titrated γD-crystallin with NaCl *in vitro* to support our assertion and included Fig S2 in Supplementary Results. Based on these results, line broadening was evident at 200 mM NaCl.

Reviewer #1: This paper has the laudable goal of understanding the nature of RNA protein interactions in E. coli lysates. But in practice they focus on the interactions of intact ribosomes with gD-crystallin. The fact that it has electrostatic interactions with a highly charged ribosome

is hardly surprising. The real question is why are these spurious interactions absent in the cell and /or managed so that they do not interfere with protein synthesis. This is not addressed in the slightest.

Quinary interactions are present in-cell as evidenced by the line broadening observed in the in-cell NMR spectrum of γD-crystallin (Fig 1B). Since quinary interactions are transient, they are not expected to interfere with high affinity interactions involved in ribosomal function. The goal of the paper is to show that it is the INTACT ribosome that mediates quinary interactions not a degraded form or "other" reactive species, and that quinary interactions are a generalized phenomenon associated with ribosomes. Consequently the effect of the interaction on activity was not considered. This is clarified in the introduction

Moreover, Gd crystallin is only enriched in human ovary cells, so it is not a particularly interesting protein. Overall, the impact of these experiments is minor and publication is not recommended.

We now included the rationale for studying γD-crystallin in *E. coli* in the first results section.

Reviewer #2: The authors tried to identify the ribosome as the major component of the quinary structure. They prove their idea using a eukaryotic protein and prokaryotic ribosomes. The similar spectral broadening in the presence of purified ribosomes and lysate suggest the ribosome as the major source of broadening. Several major concerns needed to be addressed before publication

1) The experimental data should include the HSQC spectrum in E.coli cells, it is critical to see if the degree of broadening in cells and in the purified ribosomes (the concentration is close to that in cells) are similar

The in-cell HSQC NMR spectrum of γD-crystallin in *E. coli* is now shown in Fig 1B.

2) It will be fantastic if the authors can provide the quantitative data, such as the degree of resonances broadening as the function of the purified ribosomes concentration.

The spectra of γD-crystallin with increasing amounts of ribosome are included in Fig S1. The intensities of γD-crystallin peaks as a function of ribosome concentration are included in a Table in Supplementary Results.

3) If the conclusion of the manuscript stands, the author should have more discussions on the difference between prokaryotic and eukaryotic cells and meaning for in-cell nmr field.

Based on our previous observations (ref. 1, 15-16) and our current work, we expect that in-cell NMR spectra of proteins are better resolved in eukaryotic then prokaryotic cells. It is a direct consequence of the fact that ribosome concentration in eukaryotes is about ten fold less than in prokaryotes. Because the emphasis is on the generic aspect of ribosome-protein interactions, we do not elaborate on specific differences between eukaryotic and prokaryotic ribosomes, except for their general role in regulation.
