# Supplementary material for: Intact ribosomes drive the formation of protein quinary structure
Source: PLoS One. 2020 Apr 24;15(4):e0232015. doi: 10.1371/journal.pone.0232015 (PMC7182177; doi:10.1371/journal.pone.0232015)
Supplement: Attachment — Submitted filename: renamed_64b62.pdf [file pone.0232015.s007.pdf]

Response to Reviewers' comments

We thank our reviewer for the comments.

Reviewer#2

The revised version still did not provide a quantitative analysis of resonance broadening contribution from ribosome vs other cellular components in in-cell NMR experiments, ie, at a physiological concentration of ribosome, the authors could estimate what percentage of broadening in living cells come from interacting with ribosomes for γD-crystallin since they have the in-cell and ribosome titration data. This analysis is important for the main conclusion of this manuscript "Intact ribosome particles were shown to be sufficient to mimic quinary interactions present in the crowded cytosol".

We clarified our reasoning for using *in vitro* samples to analyze the role of ribosomes in quinary interactions: "Due to the extreme heterogeneity of in-cell NMR samples and high concentration of cellular ribosomes, ~20 μM, which completely broaden the protein NMR peaks (Fig 1A and Fig 1B), direct quantitative comparison of in-cell and *in vitro* NMR spectra is not possible. Nevertheless, the observed in-cell peak broadening coincides with the presence of intact ribosomal particles in cell lysates and *in vitro*, and is consistent with intact ribosomes as the major interactor that gives rise to protein quinary interactions (Fig 3)." (Lines 176-182). Furthermore, the quantitative comparison of peak broadening is made between *in vitro* samples of γD-crystallin with diluted lysate and with purified ribosomes (Lines 131-135 and Fig S2, Fig 3C, Fig 3D, and Fig 3E).
